# Reduced interneuronal dendritic arborization in CA1 but not in CA3 region of mice subjected to chronic mild stress

**DOI:** 10.1002/brb3.534

**Published:** 2016-12-14

**Authors:** Javier Gilabert‐Juan, Clara Bueno‐Fernandez, Esther Castillo‐Gomez, Juan Nacher

**Affiliations:** ^1^ Neurobiology UnitProgram in Neurosciences and Interdisciplinary Research Structure for Biotechnology and Biomedicine (BIOTECMED)Universitat de ValènciaBurjassotSpain; ^2^ Genetics DepartmentUniversitat de ValènciaBurjassotSpain; ^3^ CIBERSAM: Spanish National Network for Research in Mental HealthBurjassotSpain; ^4^ Fundación Investigación Hospital Clínico de ValenciaINCLIVABurjassotSpain

**Keywords:** Hippocampus, Inhibition, GAD67, PSA‐NCAM, structural plasticity

## Abstract

**Introduction:**

Chronic stress induces dendritic atrophy and decreases spine density in excitatory hippocampal neurons, although there is also ample evidence indicating that the GABAergic system is altered in the hippocampus after this aversive experience. Chronic stress causes dendritic remodeling both in excitatory neurons and interneurons in the medial prefrontal cortex and the amygdala.

**Methods:**

In order to know whether it also has an impact on the structure and neurotransmission of hippocampal interneurons, we have analyzed the dendritic arborization, spine density, and the expression of markers of inhibitory synapses and plasticity in the hippocampus of mice submitted to 21 days of mild restrain stress. The analyses were performed in GIN mice, a strain that displays EGFP‐labeled interneurons.

**Results:**

We observed a significant decrease in the dendritic arborization of interneurons in the CA1 region, which did not occur in those in CA3. We found neither changes in dendritic spine density in these regions nor alterations in the number of EGFP‐positive interneurons. Nevertheless, the expression of glutamic acid decarboxylase 67 was reduced in different layers of CA1 and CA3 regions of the hippocampus. No significant changes were found in the expression of the polysialylated form of the neural cell adhesion molecule (PSA‐NCAM) or synaptophysin.

**Conclusions:**

Chronic stress reduces the interneuronal dendritic arborization in CA1 region of the hippocampus but not in CA3.

## 1 Introduction

Chronic stress alters neuronal structure in different brain regions. Several studies on animal models have demonstrated that this aversive experience causes dendritic atrophy and decreases spine density in principal neurons of the hippocampus and the medial prefrontal cortex (mPFC) (Cook & Wellman, 2004; Radley et al., 2006, 2004; Sousa, Lukoyanov, Madeira, Almeida, & Paula‐Barbosa, 2000; Watanabe, Gould, & McEwen, 1992). By contrast, in the orbitofrontal cortex or the basolateral amygdala, the effect of the chronic stress in principal neurons is the opposite, increasing spine density and dendritic arborization (Liston et al., 2006; Vyas, Mitra, Shankaranarayana Rao, & Chattarji, 2002).

In addition to these structural studies focused on principal neurons, during recent years our laboratory has analyzed the effects of chronic stress on the remodeling of interneurons. We have focused our studies on a subpopulation of spiny interneurons, which mainly express somatostatin and can be classified as Martinotti cells (Oliva, Jiang, Lam, Smith, & Swann, 2000). We have shown that chronic mild stress induces decreases in the dendritic arborization of interneurons in the lateral and basolateral amygdala of adult mice (Gilabert‐Juan, Castillo‐Gomez, Pérez‐Rando, Moltó, & Nacher, 2011) and increases this parameter in interneurons of the mPFC (Gilabert‐Juan, Castillo‐Gomez, Guirado, Moltó, & Nacher, 2013). Interestingly, these changes occur in the opposite direction to those observed in principal neurons of these regions (Radley et al., 2004; Vyas et al., 2002). However, despite the dramatic effects of chronic stress on the structure of principal neurons in the hippocampus, there are no studies yet on its impact on the structure of hippocampal interneurons. The development of research on this subject is specially interesting because different lines of evidence indicate that chronic stress has also an important impact on other features of inhibitory hippocampal neurons. This aversive experience reduces the density of different populations of interneurons in distinct hippocampal subregions (Czeh et al., 2005; Czéh et al., 2015; Hu, Zhang, Czéh, Flügge, & Zhang, 2010).

The main objective of this study was to identify structural alterations (dendritic arborization and spine density) in GABAergic interneurons of the hippocampus of chronically stressed mice and to relate these changes to alterations in the expression of molecules implicated in inhibitory neurotransmission and neuronal plasticity, such as the polysialylated form of the neural cell adhesion molecule (PSA‐NCAM), the glutamic acid decarboxylase 67 (GAD67), or the marker of synapses, synaptophysin (SYP). In the adult hippocampus PSA‐NCAM is strongly expressed in immature granule neurons, but our laboratory also found this molecule in a subpopulation of hippocampal interneurons (Nacher, Blasco‐Ibáñez, & McEwen, 2002). These interneurons have reduced dendritic arborization and spine density, as well as decreased density of perisomatic innervation when compared with interneurons lacking PSA‐NCAM expression (Gómez‐Climent et al., 2011). Consequently, this molecule may play an insulating role, modulating the connectivity of these inhibitory cells.

## 2 Material and Methods

Thirteen young‐adult (3‐month‐old) male GIN mice (EGFP‐expressing inhibitory neurons, Tg(GadGFP‐45704Swn)), in which EGFP expression is under the GAD67 promoter, were used in this experiment (Jackson laboratories, Bar Harbor, ME, USA). The animals were housed in standard conditions, as described previously (Gilabert‐Juan et al., 2013). All animal experimentation was conducted in accordance with the Directive 2010/63/EU of the European Parliament and of the Council of 22 September 2010 on the protection of animals used for scientific purposes and was approved by the Committee on Bioethics of the Universitat de València.

The chronic mild restraint stress procedure and histological techniques were performed as previously described (Gilabert‐Juan et al., 2013, 2011). Briefly, mice (*n* = 7) were immobilized for 1 hr per day for 21 days (from 11 to 12 a.m.) in transparent 50‐ml plastic conical tubes with many air holes to allow ventilation. Control animals (*n* = 6) were handled daily, but were left undisturbed in their cages after <1 min.

Mice were euthanized 24 hr after the last stress session in a random order, in a different room than the one in which restraints were carried out. Animals were perfused transcardially with 4% paraformaldehyde in phosphate buffer (0.1 M, pH 7.2). The right hemisphere was cryoprotected in a 30% sucrose solution in PB and cut in a sliding microtome at 50 μm. These sections were destined for immunohistochemical analyses. The contralateral hemisphere was cut in 100 μm sections with a vibratome and the resulting sections were used to analyze dendritic spine density on GFP expressing interneurons.

Immunohistochemistry for conventional light microscopy was performed in three subseries (50‐μm‐thick sections) from each animal and anti‐PSA‐NCAM (AbCys, 1:700, Cortabouef, France), anti‐GAD67 (Chemicon, 1:500, Millipore EMB, Bedford, MA), and anti‐SYP (Sigma, 1:200, Sigma‐Aldrich, St. Louis, MO) antibodies were used. In order to amplify the EGFP fluorescent signal in interneurons destined to morphological analysis (dendritic spine density and dendritic arborization), a simple fluorescent immunohistochemistry against EGFP was performed with anti‐GFP antibody (Millipore, 1:1,000, Millipore EMB, Bedford, MA).

Sections stained by immunohistochemistry for conventional microscopy were examined with an Olympus CX41 microscope under bright‐field illumination, homogeneously lighted and digitalized using a CCD camera. Photographs to the different areas and layers were taken at 20× magnification. Gray levels were measured using Image J software (NIH).

Dendritic arborization and spine density were studied in neurons located in the oriens layer of CA1 and CA3 regions. We randomly selected only six isolated GAD‐GFP neurons per animal and region as previously described (Gilabert‐Juan et al., 2013, 2011; Gómez‐Climent et al., 2011). In order to be analyzed, GAD‐GFP‐expressing cells had to fulfill the following features: (1) the cell must not show any truncated dendrites, (2) the dendritic arbor of the cell must show at least a process with a length greater than 150 μm, and (3) the soma must be located at least 30 μm deep from the surface of the tissue. The stacks obtained were then processed using FIJI (ImageJ) software (NIH) in order to render 3D reconstructions. The neurons were traced using the “Simple neurite tracer” ImageJ plugin, which also allowed us to analyze their Sholl profile in 3D (Longair et al., 2011). Spines were defined as any kind of protrusion found in a dendrite and were quantified in three successive segments of 50 μm distances up to a total length of 150 μm. Both structural parameters were studied with a confocal microscope (Leica, SPE, Leica Microsystems, Wetzlar, Germany), which allowed us to follow in depth the dendrites unbiasedly.

The number of neuronal somata‐expressing GAD67‐EGFP covering the total hippocampus and distinguishing by layers (oriens, lucidum, and radiatum) and regions (CA1 and CA3) was estimated using a modified version of the fractionator method (West et al., 1991), as described before (Varea et al., 2007; Castillo‐Gómez et al., 2011). Briefly, the fractionator sampling scheme refers to the methodology of examining one of every six brain sections. One from six systematic‐random series of sections covering the whole rostral to caudal extension of hippocampus was viewed on an Olympus BX61 fluorescent microscope for GAD67‐EGFP cells. Cell somata were identified and counted with a 40× objective. Cells appearing in the upper focal plane were omitted to prevent counting cell caps.

Means were determined for each experimental group and data were analyzed by Student's *t*‐test statistical analysis using the SPSS statistics software (IBM, version 19, IBM‐Deutschland GmbH, Munich, Germany).

## 3 Results

**Figure 1:**
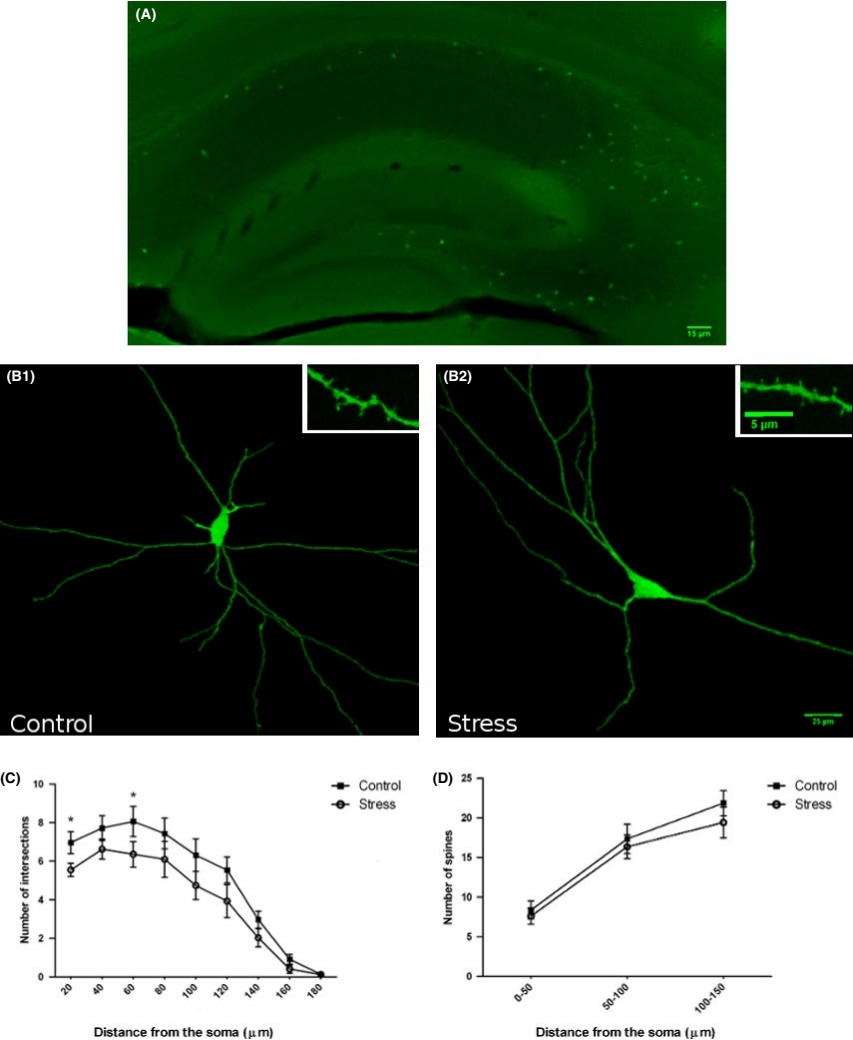
(A) Regional eGFP cells labeling pattern and distribution in the hippocampus. (B) 2D projections of GAD67‐EGFP‐expressing interneurons in the Oriens layer of the CA1 region of control (B1) and chronically stressed animals (B2). *Insets* in A1 and A2 show high‐magnification views of dendritic segments bearing spines. (C) Sholl analysis of GAD67‐EGFP‐expressing interneurons, showing the number of intersections per 20 μm dendritic radial unit distance from the soma. Unpaired Student *t*‐test showed a significant reduction in dendritic arborization in stressed mice (*p* < .05) in the 20‐ and 60‐μm segments. (D) Spine density was determined in three 50‐μm‐length segments located 0–50, 50–100, and 100–150 μm from the soma, respectively. Unpaired Student *t*‐test showed no statistically significant differences in any of the segments analyzed. *Scale bar* 15 μm for A1, 25 μm for B1 and B2; 5 μm for *insets*

**Figure 2:**
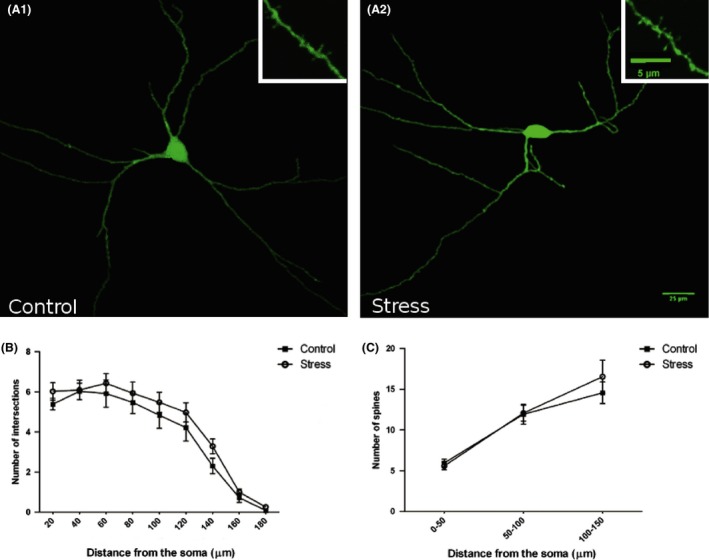
2D projections of GAD67‐EGFP‐expressing interneurons in the Oriens layer of the CA3 region of control (A1) and chronically stressed animals (A2). *Insets* in A1 and A2 show high‐magnification views of dendritic spines of GAD67‐EGFP‐expressing interneurons. (B) Sholl analysis of GAD67‐EGFP‐expressing interneurons, showing the number of intersections per 20‐μm dendritic radial unit distance from the soma. Unpaired Student *t*‐test showed no statistically significant differences. (C) Spine density was determined in three 50‐μm‐length segments located 0–50, 50–100, and 100–150 μm from the soma, respectively. Unpaired Student *t*‐test showed no statistically significant differences in any of the segments analyzed. *Scale bar* 25 μm for A1 and A2; 5 μm for *insets*

Sholl analysis showed decreased dendritic arborization in GAD67‐EGFP expressing neurons in the CA1 region of stressed mice (Figure 1), but not in those of CA3 (Figure 2). These differences in CA1 were statistically significant in two of the 20‐μm‐length segments of distance from the soma that were analyzed: 0–20 μm segment (*p *=* *.0034), 40–60 μm segment (*p *=* *.0493), and a trend toward a reduction was observed in the rest of segments. No differences in spine density were found in any segment of the interneurons of CA1 or CA3 regions (Figures 1, 2).

No significant differences in the number of neuronal somata‐expressing GAD67‐EGFP were observed in any region or layer of interest, indicating that the number of this subpopulation of interneurons is not altered under stress conditions.

Quantification of neuropil immunoreactivity was performed for each immunostaining (GAD67, PSA‐NCAM, and SYP) as previously described (Gilabert‐Juan et al., 2011). Different layers of CA1 and CA3 regions were selected in order to measure immunoreactivity (Table 1). In the stressed mice, GAD67 expression was reduced significantly in the stratum lacunosum‐moleculare of CA1 (*p *=* *.048) and in the strata lucidum (*p *=* *.004) and radiatum (*p *=* *.005) of CA3. No significant changes induced by stress were observed for SYP or PSA‐NCAM expression in the different layers and regions studied. We also analyzed the neuropil immunoreactivity in the different layers of the dentate gyrus, but we did not find any significant difference induced by stress (data not shown).

**Table 1:** Staining intensity of GAD67, PSA, and SYP molecules in the different layers of the hippocampus measured in arbitrary units

Region	Layer	Control	Stress	*p*‐value
GAD67				
CA1	Oriens	121.633 ± 8.720	116.366 ± 3.917	.220
	Pyramidal	147.966 ± 9.196	140.533 ± 3.221	.091
	Radiatum	116.933 ± 7.216	113.933 ± 7.216	.399
	Lacunosum‐Molec.	128.466 ± 9.931	117.766 ± 6.033	.048*
CA3	Oriens	137.100 ± 14.419	135.133 ± 7.934	.778
	Pyramidal	151.366 ± 9.341	144.400 ± 3.530	.118
	Lucidum	171.266 ± 4.679	163.000 ± 2.561	.004*
	Radiatum	132.900 ± 5.274	123.666 ± 3.545	.005*
	Lacunosum‐Molec.	140.200 ± 3.008	138.543 ± 4.853	.786
PSA‐NCAM				
CA1	Oriens	121.006 ± 7.258	115.857 ± 4.350	.138
	Pyramidal	133.800 ± 3.706	126.943 ± 2.318	.134
	Radiatum	119.266 ± 6.180	116.371 ± 4.668	.375
	Lacunosum‐Molec.	133.933 ± 4.668	136.771 ± 15.247	.735
CA3	Oriens	137.400 ± 7.767	134.914 ± 7.615	.573
	Pyramidal	127.650 ± 1.877	128.029 ± 2.692	.913
	Lucidum	175.566 ± 11.608	165.687 ± 5.728	.099
	Radiatum	124.733 ± 7.355	121.228 ± 1.682	.301
	Lacunosum‐Molec.	157.400 ± 3.042	150.314 ± 1.757	.063
SYP				
CA1	Oriens	142.466 ± 10.379	136.00 ± 12.373	.334
	Pyramidal	85.500 ± 4.809	82.571 ± 4.176	.653
	Radiatum	132.266 ± 12.757	125.314 ± 13.485	.363
	Lacunosum‐Molec.	102.700 ± 17.850	94.742 ± 13.816	.384
CA3	Oriens	145.880 ± 3.537	141.914 ± 12.243	.503
	Pyramidal	89.067 ± 5.739	84.171 ± 4.255	.499
	Lucidum	143.433 ± 10.074	140 ± 7.185	.555
	Radiatum	132.600 ± 11.625	125.685 ± 11.733	.310
	Lacunosum‐Molec.	132.567 ± 5.387	131.000 ± 3.821	.8129

GAD67, glutamic acid decarboxylase 67; PSA‐NCAM, polysialylated form of the neural cell adhesion molecule; SYP, synaptophysin.

Significant values (*p*≤0.05) are indicated by bold characters and an asterisk.

## 4 Discussion

The animals used in this study did not present stress‐induced changes in their body weight (Gilabert‐Juan et al., 2011), contrary to what it has been found in other strains (Chmielarz et al., 2006). Although there are no other experiments of chronic restrain stress using this strain, it is possible that it may be particularly resilient to weight changes, as other chronic stressors, such as social isolation, also failed to induce changes in body weight (Volden et al., 2013). Future studies should measure corticosterone levels and adrenal/thymus weights in order to reveal physiological changes associated with the stress‐induced neuronal plasticity.

Our results show a reduction in the dendritic arborization of interneurons in the CA1, but not in CA3, after chronic stress. This chronic restraint stress paradigm (1 hr per day, 21 days) should be considered mild. In fact, previous studies have demonstrated that in rats 2 hr of restraint per day are not enough to induce dendritic remodeling in pyramidal hippocampal neurons (McLaughlin, Gomez, Baran, & Conrad, 2007). However, similar restraint durations (2–3 hr) are able to induce morphological, neurochemical, and behavioral changes in mice (Qin, Xia, Huang, & Smith, 2011; Satoh, Tada, & Matsuhisa, 2011) and our previous experiments using the present strain and stress paradigm showed changes in the structure of amygdaloid and hippocampal interneurons (Gilabert‐Juan et al., 2013, 2011). The EGFP‐labeled cells in the CA1 region of GIN mice are O‐LM interneurons, which project to the distal apical dendrites of pyramidal neurons in the stratum lacunosum‐moleculare (Oliva et al., 2000). Previous studies in rats have revealed that chronic stress can induce atrophy of the apical dendritic tree of CA1 pyramidal neurons, including a retraction of their dendritic terminal segments (Sousa et al., 2000). Similar results have been obtained in the CA1 region of some mice strains subjected to chronic stress, where apical dendritic atrophy and spine density reduction have been observed (Christian, Miracle, Wellman, & Nakazawa, 2011; Magariños et al., 2011; Pawlak et al., 2005). It is possible that the reduction in dendritic arborization in the O‐LM interneurons may also result in a decrease in the density or alterations in the neurotransmission of the synaptic contacts that they make on the distal dendrites of pyramidal neurons. In the opposite direction, the atrophy of the distal arbor of pyramidal neurons may also induce reductions in the number of synaptic contacts made by O‐LM interneurons or their function and this has an impact on their dendritic arborization. Future experiments should explore in detail the time course of the neuronal remodeling induced by stress in both principal neurons and interneurons. Notwithstanding, the reduction in GAD67 expression that we have observed in the CA1 stratum lacunosum‐moleculare after stress is consistent with these putative reductions in the input that O‐LM cells send to the distal dendrites of pyramidal neurons. No changes in GAD67 expression were detected in the stratum lacunosum‐moleculare of CA3, which may be explained, at least partially, by the lack of structural remodeling in CA3 O‐LM interneurons. However, reductions in GAD67 expression have been observed in the strata lucidum and radiatum, which most likely are due to changes in other interneuronal subpopulations, such as the mossy fiber‐associated (MFA) or the calbindin‐expressing multipolar interneurons of CA3, among others (Freund & Buzsáki, 1996; Vida & Frotscher, 2000). It is also possible that at least part of this reduction could be due to cell death. Although there is no evidence of neuronal degeneration in this hippocampal region after this paradigm of chronic stress, a 3‐week treatment with corticosterone produced a decrease in the density of cells in the CA3 but not in the CA1 of adult rats (Sapolsky, Krey, & McEwen, 1985). The previous literature on the expression of this marker of inhibitory neurotransmission in the CA1 has described increases after chronic social stress (Makinson, Lundgren, Seroogy, & Herman, 2015), but not after chronic variable intermittent stress (Bowers, Cullinan, & Herman, 1998). However, these studies measured mRNA expression and were not focused in a particular layer.

Our results on the analysis of PSA‐NCAM expression are similar to those found previously in the mPFC of GIN mice, where chronic stress did not induce changes (Gilabert‐Juan et al., 2013). Previous studies in rats have found increases in the hippocampal expression of this plasticity‐related molecule after chronic restrain stress (Pham, Nacher, Hof, & McEwen, 2003; Sandi, Merino, Cordero, Touyarot, & Venero, 2001). However, the daily restrain period used was considerably longer in rats than in our mice. Moreover, the increased expression of PSA‐NCAM in rats appears to be associated mainly with changes in granule neurons and their projections to CA3 stratum lucidum (Pham et al., 2003).

Our study adds to the growing evidence that hippocampal inhibitory networks are also the targets of chronic stress. A negative impact of this aversive experience on the density of interneurons‐expressing parvalbumin, calretinin, and also in those expressing neuropeptide Y and somatostatin has been recently described in rats (Czéh et al., 2015). Although most of EGFP‐labeled interneurons in the hippocampus of GIN mice express somatostatin, we have failed to find decreases in the number of these interneurons in our model. This discrepancy may be explained by the longer exposure to stressor in the Czéh et al. (2015) study (9 weeks vs. 3 weeks in our study), as well as by species differences in the response to chronic stressors.

## Conflict of Interest

All authors declare no conflict of interest.

## Funding Information

Spanish Ministry of Economy and Competitiveness. Grant Number: BFU2012‐32512, Generalitat Valenciana Prometeo Excellence Program. Grant Number: PROMETEO2013/069 and the Fundación Alicia Koplowitz to JN.
